# Apigenin inhibits angiogenesis in retinal microvascular endothelial cells through regulating of the miR-140-5p/HDAC3-mediated PTEN/PI3K/AKT pathway

**DOI:** 10.1186/s12886-023-03046-5

**Published:** 2023-07-06

**Authors:** Chaojun Fu, Jun Peng, Yanjun Ling, Hongqing Zhao, Yongwang Zhao, Xiuli Zhang, Min Ai, Qinghua Peng, Yuhui Qin

**Affiliations:** 1 Hunan Provincial Key Laboratory for the Prevention and Treatment of Ophthalmology and Otolaryngology Diseases With Chinese Medicine, Hunan University of Chinese Medicine, Hanpu Rd., Yuelu District, Changsha, 410208 Hunan China; 2 Hunan Engineering Technological Research Center for the Prevention and Treatment of Otolaryngologic Disease and Protection of Visual Function With Chinese Medicine, Changsha, 410208 Hunan China; 3 Ophthalmology Department, The First Hospital of Hunan University of Chinese Medicine, Shaoshan Rd., Yuhua District, Changsha, 410007 Hunan China; 4 Institute of Chinese Medicine of Hunan Province, Lushan Rd., Yuelu District, Changsha, 410006 Hunan China; 5 Ophthalmology Department, Songjiang Branch of the First People’s Hospital affiliated to Shanghai Jiao Tong University, Zhongshan Zhong Rd., Songjiang District, Shanghai, 201699 China

**Keywords:** Apigenin, miR-140-5p/HDAC3, PTEN/PI3K/AKT, Angiogenesis, Diabetic retinopathy

## Abstract

**Background:**

Diabetic retinopathy (DR) is a common cause of visual impairment. Apigenin has been shown to have antiangiogenic effects in various diseases. Our study aimed to investigate the role of apigenin in DR and elucidate the underlying mechanism.

**Methods:**

Human retinal microvascular endothelial cells (HRMECs) were exposed to high glucose (HG) to establish a DR model. HRMECs were treated with apigenin. Then we knocked down or overexpressed miR-140-5p and HDAC3, and added PI3K/AKT inhibitor LY294002. The expression levels of miR-140-5p, HDAC3, and PTEN were measured using qRT-PCR. Western blot analysis was performed to assess the expression of HDAC3, PTEN, and PI3K/AKT pathway-related proteins. Finally, cell proliferation and migration were evaluated using MTT, wound-healing assay, and transwell assay, while angiogenesis was examined using the tube formation assay.

**Results:**

HG treatment resulted in reduced miR-140-5p expression and overexpression of miR-140-5p suppressed proliferation, migration, and angiogenesis of the HG-induced HRMECs. Apigenin treatment significantly restored the decreased level of miR-140-5p caused by HG treatment and inhibited proliferation, migration, and angiogenesis of the HG-induced HRMECs by upregulating miR-140-5p. Moreover, miR-140-5p targeted HDAC3, and overexpression of miR-140-5p reversed the HG-inducted upregulation of HDAC3 expression. HDAC3 was found to bind to the promoter region of PTEN, inhibiting its expression. Knockdown of HDAC3 suppressed the PI3K/AKT pathway by elevating PTEN expression. Furthermore, apigenin inhibited angiogenesis in DR cell models through the regulating of the miR-140-5p/HDAC3-mediated PTEN/PI3K/AKT pathway.

**Conclusions:**

Apigenin effectively suppressed angiogenesis in HG-induced HRMECs by modulating the miR-140-5p/HDAC3-mediated PTEN/PI3K/AKT pathway. Our study may contribute to the development of novel therapeutic approaches and identification of potential targets for the treatment of DR.

**Supplementary Information:**

The online version contains supplementary material available at 10.1186/s12886-023-03046-5.

## Highlights

1. Overexpression of miR-140-5p suppressed proliferation, migration, and angiogenesis of HG-induced HRMECs.

2. Apigenin suppressed HG-induced HRMECs proliferation, migration, and angiogenesis by increased the expression of miR-140-5p.

3. miR-140-5p targeted HDAC3.

4. Knockdown HDAC3 repressed PI3K/AKT pathway via elevating PTEN expression.

5. In a diabetic retinopathy (DR) cell model, apigenin can inhibit angiogenesis by regulating the miR-140-5p/HDAC3-mediated PTEN/PI3K/AKT pathway.

**Supplementary Information:**

The online version contains supplementary material available at 10.1186/s12886-023-03046-5.

## Introduction

Diabetic retinopathy (DR) is a microvascular disease of the retina that affects almost all diabetic patients [1]. DR is characterized by symptoms such as blurred vision, floaters, and vision distortion, which can progress to partial or total vision loss in severe cases [2]. Current treatment options for advanced vision loss in DR include intravitreal anti-vascular endothelial growth factor (VEGF) drugs, laser therapy, vitrectomy, and glucocorticoids [3]. However, the precise mechanism underlying abnormal angiogenesis in endothelial cells, which is a key aspect of DR [4], is still unclear. Therefore, gaining insights into the biochemical changes and molecular events associated with DR is crucial in the quest for novel and effective therapeutic approaches.

MicroRNAs (miRNAs) are small non-coding RNAs that play a role in the pathological mechanism of DR [5]. Among them, miR-140-5p has been found to be upregulated in exosomes, leading to the inhibition of angiogenesis in intervertebral disk degeneration through the regulation of the Wnt/β-catenin pathway [6]. In addition, miR-140-5p has demonstrated its ability to target VEGF-A, thereby inhibiting invasion and angiogenesis in laryngeal cancer cells [7], indicating its potential as an antiangiogenic factor. However, the specific function of miR-140-5p function in DR remains unclear.

Apigenin, a flavonoid found in edible plants, has been shown to possess antiangiogenic effects. It has been reported to inhibit angiogenesis in ovarian tumors and human lung cancer by reducing the expression of HIF-1α and VEGF [8, 9]. Notably, apigenin has an antiangiogenic effect and could increase the levels of miR-140-5p [10]. Nevertheless, the functional relationship between apigenin and miR-140-5p in the context of DR has not yet been investigated.

Histone deacetylase 3 (HDAC3) is a member of histone deacetylase family and exerts a crucial role in the occurrence and development of malignant tumors, particularly in proliferation, apoptosis, angiogenesis, metastasis, and anti-tumor drug resistance [11]. Studies have indicated upregulation of HDAC3 in diabetes [12], and increased activity of HDACs in DR [13]. Lu JM et al. reported that overexpressing miR-21in DR rats may suppress PTEN levels, thus activating the PI3K/Akt/VEGF pathway, stimulating RVEC activity and promoting angiogenesis [14]. MiR-19a can also target and bind to the PTEN protein, modulating the PI3K/Akt pathway and impacting DR progression [15]. These findings underscore the significant role of PTEN and PI3K/AKT in DR. Moreover, the regulatory relationship between HDAC3 and PTEN [16, 17] remains unclear in the context of DR.

Therefore, we conducted a preliminarily evaluation to assess the impact of apigenin on DR, and determine if the potential mechanism of action involved the regulation of the miR-140-5p/HDAC3-mediated PTEN/PI3K/AKT pathway. The findings from our research have the potential to introduce novel drugs and therapeutic targets for the treatment of DR.

## Materials and methods

### Cell treatment

293 T cells (CL-0005) and HRMECs, CP-H130) were purchased from Procell (Wuhan, China). The 293 T cells were cultured in DMEM supplement with 10% fetal bovine serum and 1% penicillin/streptomycin. HRMECs were cultured in HRMECs complete medium (CM-H130, Procell, Wuhan, China). To establish a DR cell model, HRMECs were treated with D-glucose (HG, 25 mM) for 48 h [18]. The Control group was treated with normal glucose (5 mM), while the isotonic Control group (Osm) was treated with 25 mM L-glucose for 48 h. Subsequently, HRMECs were exposed to different concentrations of apigenin (0, 5, 10, 25, 50, 100 μM) for 24 h, followed by HG treatment [19]. The cells were divided into Control, HG + Apigenin (0, 5, 10, 25, 50, 100 μM) groups. After identifying the optimal concentration, the cells were further divided into Control, MG, HG, HG + DMSO, HG + Apigenin groups. In addition, we treated HRVECs with 10 μM PI3K/AKT inhibitor LY294002 for 24 h.

### Cell transfection

GenePharma (Shanghai, China) provided si-HDAC3, miR-140-5p mimic, miR-140-5p inhibitor, and corresponding negative controls. Transfection of the aforementioned si-RNA sequences into cells was carried out using lipofectamine 3000 reagent following the provided instructions. Subsequent detection was performed after a 48 h period of transfection.

### Quantitative real-time PCR (qRT-PCR)

Total cellular RNA was extracted using Trizol (15,596,026, Thermo, USA). The cDNA reverse transcription kit (4,368,814, Invitrogen, USA) was used to convert RNA into complementary DNAs (cDNAs). Gene expression analysis was performed on the ABI 7900 system using SYBR Green qPCR Mix (HY-K0501A, MedChemExpress, USA). Using β-actin or U6 as an internal reference, gene level was calculated by 2^−ΔΔCt^ method. The primers sequences used were as follows:

- miR-140-5p (F): GGGCCAGTGGTTTTACCCTA
- miR-140-5p (R): GTCGTATCCAGTGCAGGGTCCGAGGTATTCGCACTGGATACGACCTACCA
- HDAC3 (F): CATCCAGATGTCAGCACCCG
- HDAC3 (R): AAAGTAGGCTGAAGTCCCTGC
- PTEN (F): CGACGGGAAGACAAGTTCAT
- PTEN (R): AGGTTTCCTCTGGTCCTGGT
- β-actin (F): CCCTGGAGAAGAGCTACGAG
- β-actin (R): CGTACAGGTCTTTGCGGATG
- U6 (F): CTCGCTTCGGCAGCACA
- U6 (R): AACGCTTCACGAATTTGCGT

### Western blot

Total cellular protein was extracted using RIPA lysis buffer (P0013B, Beyotime, China). The protein content of the samples was determined using the BCA protein assay kit (BL521A, Biosharp, China). The proteins were mixed with SDS-PAGE loading buffer (MB2479, Meilunbio, China) and separated by gel electrophoresis onto PVDF membranes. Subsequently, the membranes were blocked using 5% nonfat milk. Primary antibodies including HDAC3 (1:1000, ab137704, Abcam, UK), PTEN (1:1000, ab267787, Abcam, UK), PIK3 (1:1000, 4257, Cell Signaling Technology, USA), p-PIK3 (1:1000, 4228, Cell Signaling Technology, USA), AKT (1:1000, 9272, Cell Signaling Technology, USA), p-AKT (1:2000, 4060, Cell Signaling Technology, USA), and β-actin (1:1000, ab8227, Abcam, UK) were incubated with the membranes overnight at 4 °C. Then, the membranes were incubated with the respective secondary antibodies. The target protein bands were visualized using a chemiluminescence imaging system (Chemiscope6100, CLiNX, China). β-actin was used as an internal reference to normalize the protein expression levels.

### Bioinformatics prediction and dual-luciferase reporter assay

The binding sites of miR-140-5p to HDAC3 were predicted using the Starbase database (https://starbase.sysu.edu.cn/). Furthermore, the ThTFtarget database (http://bioinfo.life.hust.edu.cn/hTFtarget#!/) predicted the binding site of HDAC3 to the upstream promoter region of PTEN.

To confirm the binding of miR-140-5p to 3′UTR of HDAC3, fragments of wild-type (WT) or mutant (MUT) HDAC3 were inserted into the pmirGLO vector (E1330, Promega, USA). The recombinant vector was transfected into 293 T cells using the pofectamine 3000 reagent (L3000015, Thermo, USA), along with mimics NC and miR-140-5p mimics. Subsequently, luciferase activity was measured using the Nano-Glo dual-luciferase reporter assay.

### Chromatin immunoprecipitation (ChIP)-qPCR

The cells were fixed with 1% formalin, followed by random fragmentation of DNA to sizes ranging from 200–800 bp using ultrasound. Immunoprecipitation was performed using a specific antibody against HDAC3 or IgG to capture the target protein–DNA complex. Subsequently, the target protein–DNA complex was de-cross-linked by incubating overnight at 65 °C. To purify and elute the ChIP DNA, 100 μL of H_2_O was applied. For qPCR analysis, 2.5 μL ChIP DNA was used [20].

### MTT assay

The cells were digested, counted, and seeded into a 96-well plate (1 × 10^4^ cells/well). After culturing for the designated time according to the experimental groups, 10 μL of 5 mg/mL MTT solution was added to each well followed by incubation at 37 °C with 5% CO_2_ for 4 h. Then, a 96-well plate was then taken out, and the MTT-containing medium was carefully aspirated. Subsequently, 150 μL of DMSO was added to each well and the plate was gently shaken at room temperature for 10 min. The absorbance at 490 nm was assessed using a Bio-Tek microplate reader (MB-530, Heales, China).

### Wound-healing assay

The cells were digested and counted using trypsin. Approximately 5 × 10^5^ cells were seeded in each well of the plate. Once the cells reached a confluence of approximately 90%, they were washed once with sterile PBS and a scratch was made using a sterile pipette tip. The medium was then added to the wells. Photographs were captured at 0 h to document the initial scratch. After incubating at 37 °C with 5% CO_2_ for 24 h, pictures were recorded again.

### Transwell migration assay

Cell migration was assessed using the Transwell chamber method. Cells were suspended at 1 × 10^6^/mL in serum-free medium. A total of 100 μL of the cell suspension was added to the upper chamber of the transwell chamber (33,318,035, Corning), while 600 μL of complete medium was added to the lower chamber. The culture medium inside the chamber was discarded, and cells on the upper chamber surface were gently wiped away using a damp cotton swab. The remaining cells were fixed with 4% paraformaldehyde, stained with 0.5% crystal violet, and then washed with water. The cells on the outer surface of the upper chamber were observed and photographed under a microscope (Olympus, Japan).

### Tube formation assay

Matrigel (356234, Biocoat) was placed on an ice box. Then, 70 μL of Matrigel was added to each well of a pre-cooled 96-well plate ensuring that the wells were evenly covered. The plate was allowed to stand at 4 °C for 10 min followed by placement in a 37 °C incubator for 30 min. Then, the cells were digested using 0.25% trypsin, and cell counting was performed using a cell counting plate. Subsequently, 10,000 cells were added to each well containing the Matrigel. Then, the number of meshes, tube length, and number of nodes were calculated.

### Statistical analysis

Statistical analysis was performed using GraphPad Prism8.0 software. Measurement data were presented as mean ± standard deviation (SD). Group comparisons were analyzed using Student’s t-test. One-way ANOVA followed by Tukey’s post hoc test was applied for multiple group comparisons. A *p-*value of less than 0.05 was considered statistically significant.

## Results

### Overexpression of miR-140-5p inhibited HG-induced HRMECs proliferation, migration, and angiogenesis

**Fig. 1:**
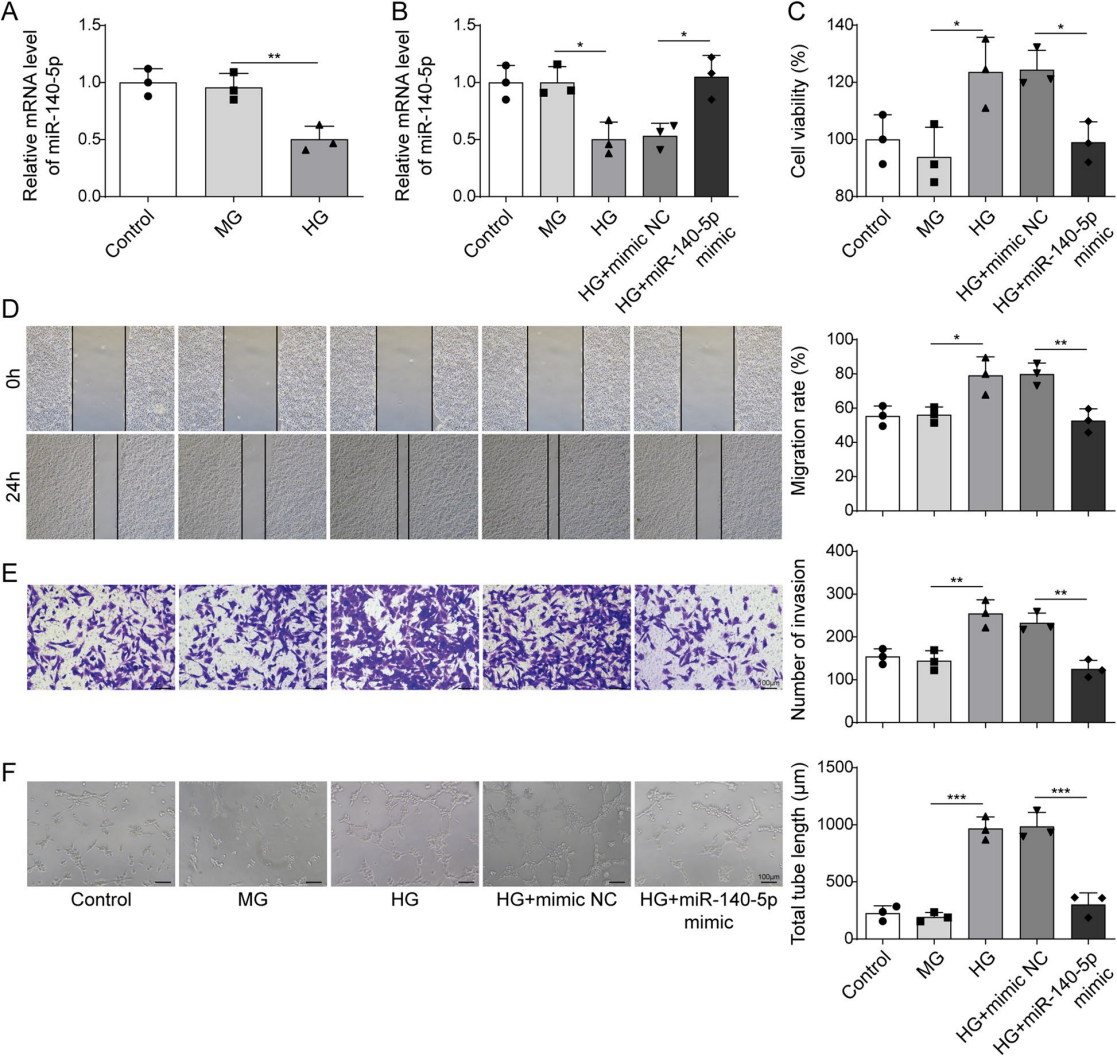
Overexpressing of miR-140-5p suppressed HG-induced HRMECs proliferation, migration, and angiogenesis. **A** After HG treatment, miR-140-5p level was determined using qRT-PCR. After overexpressing miR-140-5p and HG treatment, **B** miR-140-5p expression was examined using qRT-PCR. **C** MTT assay detection of cell proliferation. **D** Wound-healing assay assessed cell migration. **E** Transwell assay monitored cell migration. **F** Tube formation assay measured angiogenesis. *n* = 3. Data were showed as mean ± SD. *p < 0.05, ** p < 0.01, *** p < 0.001

To investigate the function of miR-140-5p, we initially established a DR cell model by treating HRMECs with HG. Then, HG treatment decreased miR-140-5p expression (Fig. 1A). Moreover, HG-induced miR-140-5p was reversed by treating cells with a miR-140-5p mimic treatment, indicating the successful overexpression of miR-140-5p (Fig. 1B). Cell function experiments showed that there was no significant difference in HRMECs activity and migration ability comparing the Control and Osm group. However, HG treatment increased HRMECs viability, and migration ability. Notably, the overexpression of miR-140-5p reversed these effects induced by HG (Fig. 1C-E). Moreover, there was no significant difference in HRMECs angiogenesis between Control and Osm group (Fig. 1F). Conversely, overexpressing miR-140-5p reversed the HG-induced elevation in HRMEC angiogenesis. Therefore, overexpression of miR-140-5p reduced proliferation, migration, and angiogenesis in HG-induced HRMECs.

### Apigenin inhibited the proliferation, migration, and angiogenesis of HG-induced HRMECs through elevating miR-140-5p

**Fig. 2:**
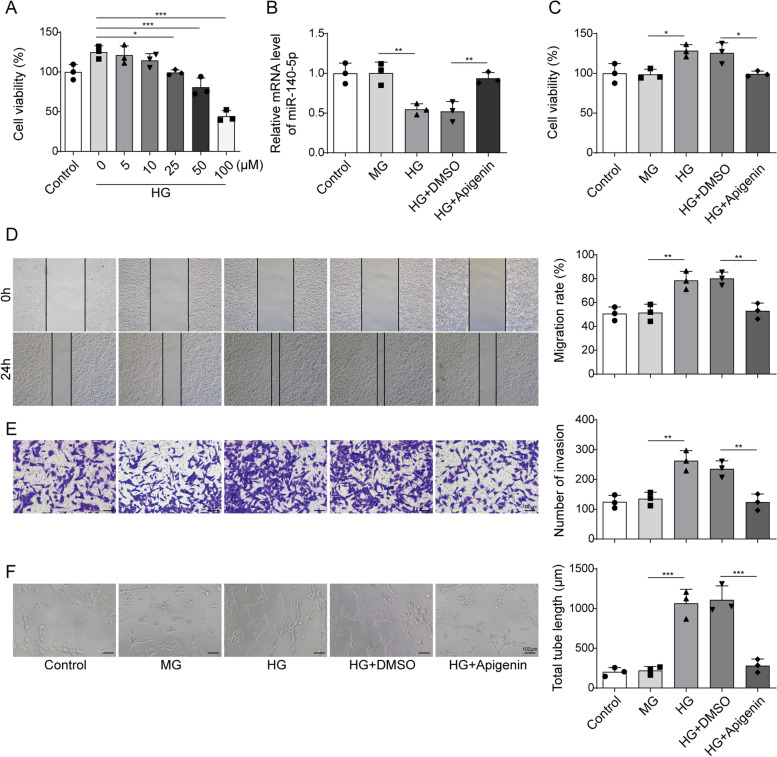
Apigenin reduced the proliferation, migration, and angiogenesis of HG-induced HRMECs through elevating miR-140-5p. **A** HRMECs were treated with various concentrations of apigenin (0, 5, 10, 25, 50, 100 μM), followed by HG treatment, and cell proliferation was tested by MTT. HG treatment was performed after apigenin treatment. **B** miR-140-5p level was assessed using qRT-PCR. **C** MTT assay tested cell proliferation. **D** Wound-healing assay analysis of cell migration. **E** Transwell assay monitored cell migration. **F** Tube formation assay tested angiogenesis. *n* = 3. Data were showed as mean ± SD. *p < 0.05, ** p < 0.01, *** p < 0.001

Apigenin nanoparticles has been shown to regulate miR-140-5p and exhibit potential therapeutic effects in renal ischemia/reperfusion [10]. We aimed to explore whether apigenin could modulate DR by regulating miR-140-5p. Initially, HRMECs were treated with varying concentrations of apigenin (0, 5, 10, 25, 50, 100 μM) following HG treatment. MTT assay revealed that HG enhanced HRMECs’ viability compared to the Control group. Moreover, the effect of apigenin on cell proliferation was dose-dependent with cell viability reaching levels similar to the Control group at dose exceeding 25 μM (Fig. 2A). Therefore, we chose 25 μM apigenin for subsequent experiments. qRT-PCR showed that miR-140-5p levels did not significantly differ between the Control and Osm groups, while apigenin treatment effectively reversed HG-induced inhibition of miR-140-5p expression (Fig. 2B). Similarly, cell function experiments showed that HG treatment increased HRMECs viability, migration ability, and angiogenesis, which were reversed by apigenin treatment (Fig. 2C–F). These results indicated that apigenin suppressed HG-induced proliferation, migration, and angiogenesis in HRMECs by elevating miR-140-5p.

### miR-140-5p targeted HDAC3

**Fig. 3:**
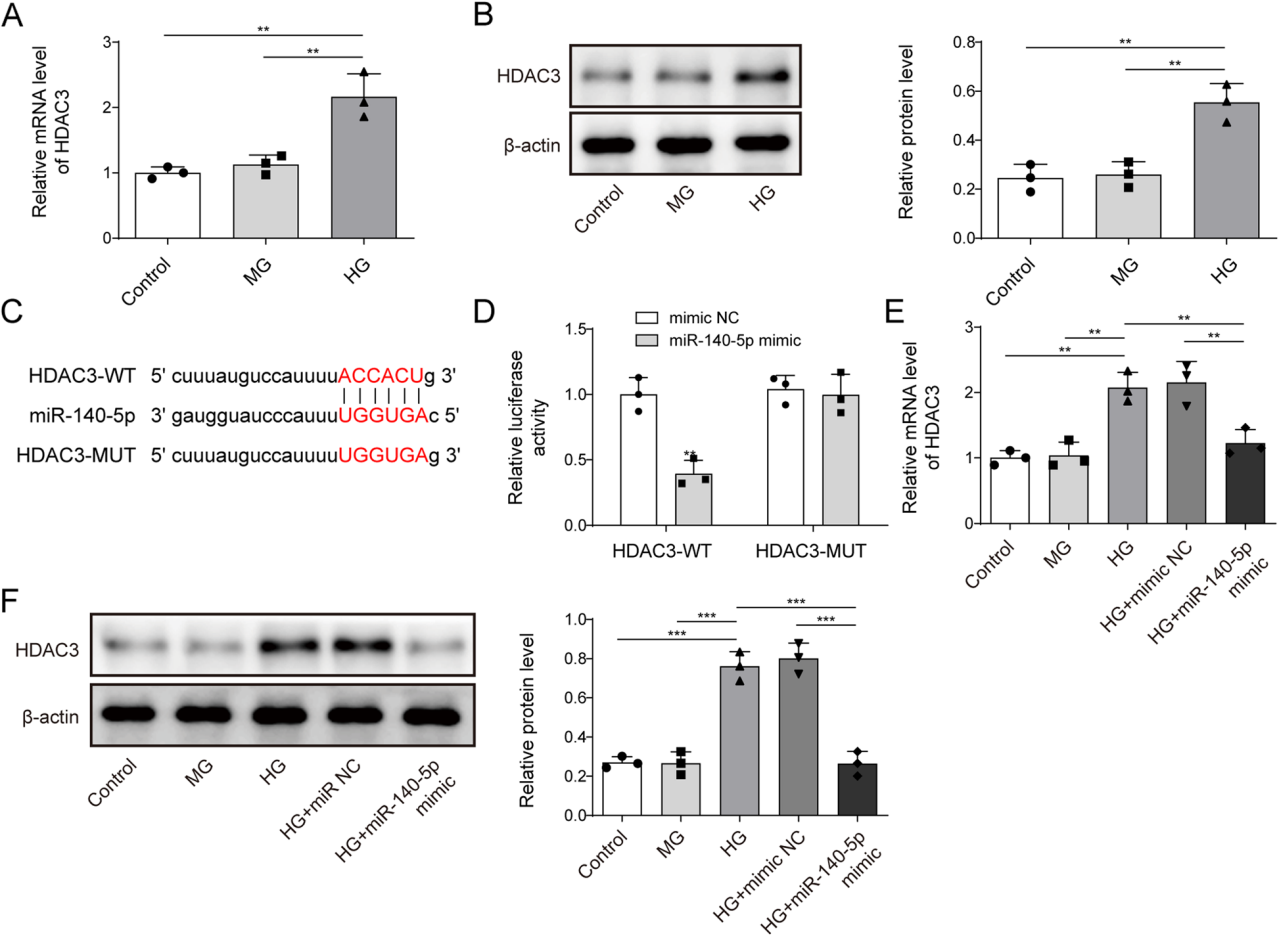
miR-140-5p targeted HDAC3. After HG treatment, HDAC3 mRNA (**A**) and protein (**B**) expressions were assessed using qRT-PCR and Western blot, respectively. **C** Starbase database predicted the binding relationship of miR-140-5p to HDAC3. **D** Dual-luciferase reporter assay verified the targeting relationship between miR-140-5p and HDAC3. After overexpression of miR-140-5p and HG treatment, HDAC3 mRNA (**E**) and protein (**F**) expressions were tested using qRT-PCR and Western blot, respectively. *n* = 3. Data were showed as mean ± SD. *p < 0.05, ** p < 0.01, *** p < 0.001

Next, we explored miR-140-5p’s target molecules. Figure 3A and B showed that HDAC3 level did not significantly differ between the Control and Osm groups, while HG treatment increased the mRNA and protein levels of HDAC3 (Fig. 3A–B). Analysis from Starbase database analysis suggested a potential targeting relationship between miR-140-5p and HDAC3 (Fig. 3C). Dual-luciferase experiments confirmed the targeting relationship, with the miR-140-5p mimic reducing luciferase activity in the WT HDAC3 3'UTR group but having no effect on the mutant group (Fig. 3D). In addition, HG treatment increased HDAC3 expression, which was significantly suppressed by the overexpression of miR-140-5p (Fig. 3E and F). Collectively, miR-140-5p targeted HDAC3 and inhibited its expression.

### Knocking down HDAC3 repressed PI3K/AKT pathway through elevating PTEN expression

**Fig. 4:**
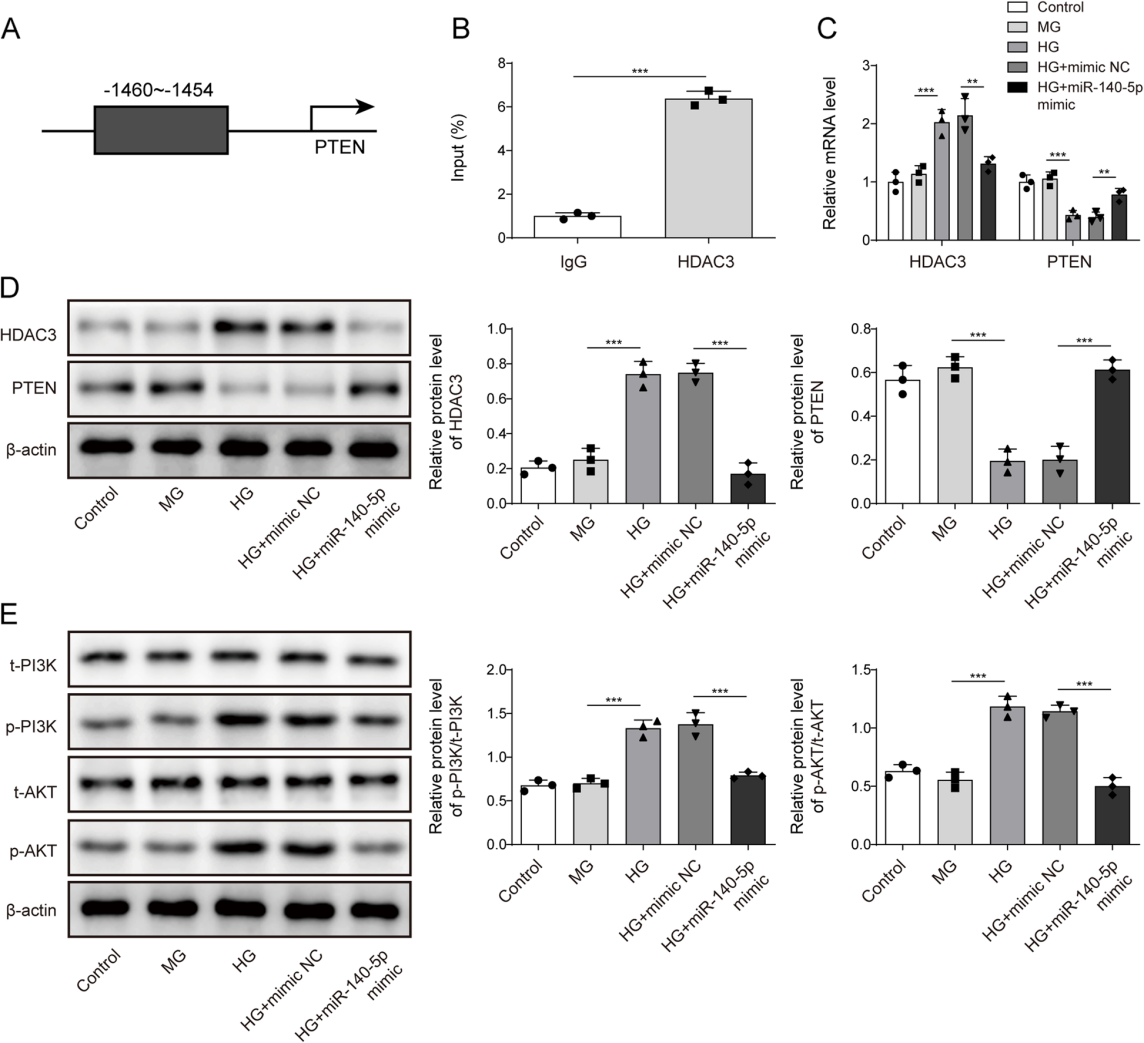
Knockdown HDAC3 repressed PI3K/AKT pathway by elevating PTEN expression. **A** The hTFtarget database predicted targeted binding relationship between HDAC3 and the upstream promoter region of PTEN. **B** ChIP-qPCR experiments verified the interaction between HDAC3 and the upstream promoter region of PTEN. After knockdown of HDAC3 and HG treatment, HDAC3 and PTEN mRNA (**C**) and protein (**D**) levels were tested using qRT-PCR and Western blot, respectively. **E** Western blot detection of PI3K, AKT, p-PI3K, p-AKT expression. *n* = 3. Data were showed as mean ± SD. *p < 0.05, ** p < 0.01, *** p < 0.001

Previous studies have shown that HDAC3 has a regulatory relationship with PTEN [16, 17]. Through analysis of the hTFtarget database, we identified a targeted binding relationship between HDAC3 and the upstream promoter region of PTEN (Fig. 4A). ChIP-qPCR results confirmed the binding of HDAC3 to the PTEN promoter region (Fig. 4B). Next, we performed aHDAC3 knock down and evaluated the expression of HDAC3 and PTEN. As shown in Fig. 4C and D, si-HDAC3 treatment neutralized the HG-induced elevation in HDAC3 levels and repression in PTEN levels. Furthermore, we observed that HG induction resulted in the activation of the PI3K/AKT pathway, which was mitigated by HDAC3 knock down (Fig. 4E). Overall, the knock down of HDAC3 repressed the PI3K/AKT pathway by increasing PTEN levels.

### Apigenin suppressed angiogenesis in DR cell model through regulating miR-140-5p/HDAC3-mediated PTEN/PI3K/AKT pathway

**Fig. 5:**
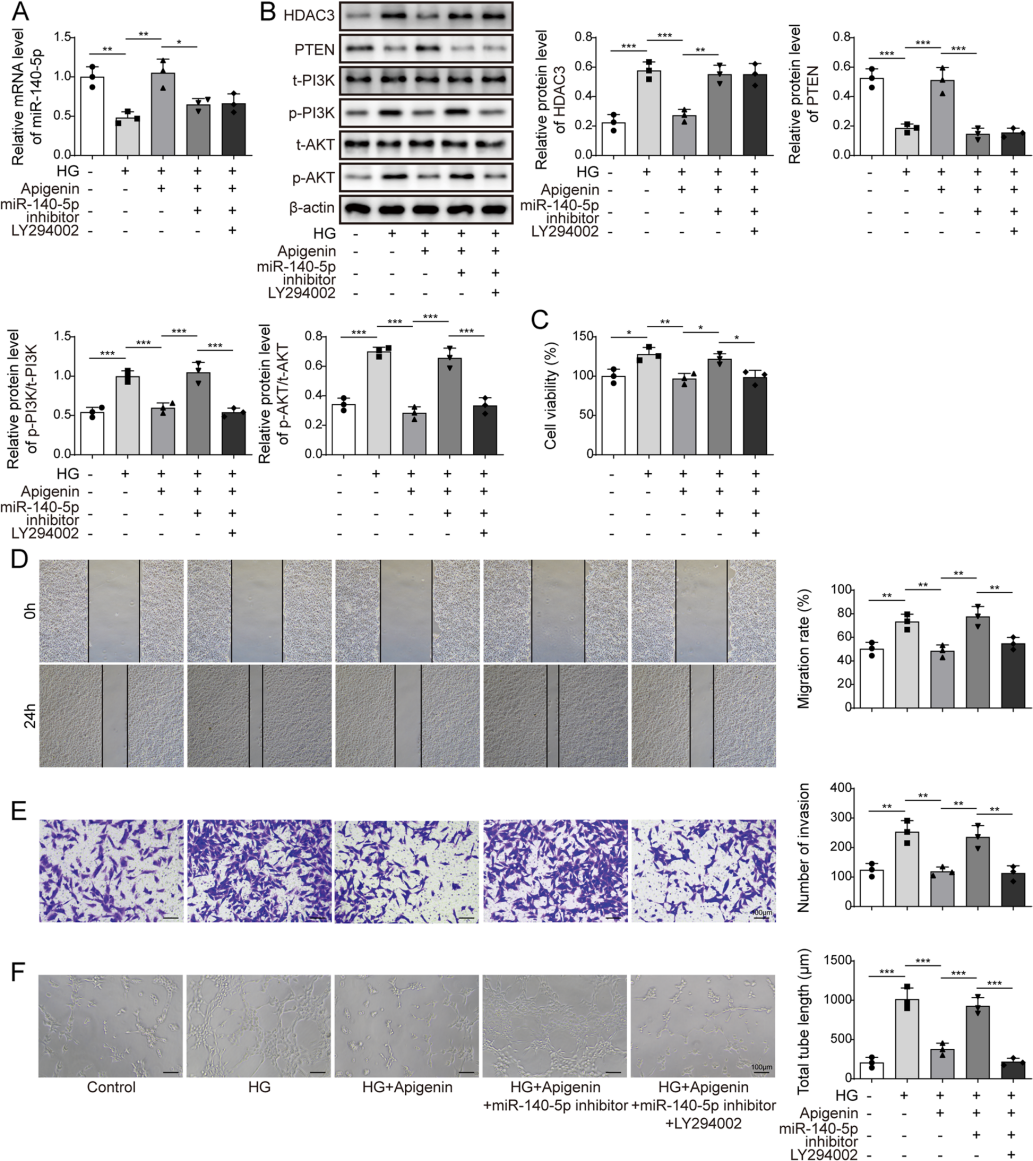
Apigenin inhibited angiogenesis in DR cell model through regulation of miR-140-5p/HDAC3-mediated PTEN/PI3K/AKT pathway. After depletion of miR-140-5p and PI3K/AKT concurrently with apigenin treatment in HG-induced HRMECs, **A** qRT-PCR detection of miR-140-5p expression. **B** Western blot detection of HDAC3, PTEN, PI3K, AKT, p-PI3K, p-AKT expression. **C** Cell proliferation analysis using MTT assay. **D** Wound-healing assay measured cell migration. **E** Transwell analysis of cell migration. **F** Tube formation assay tested angiogenesis. *n* = 3. Data were showed as mean ± SD. *p < 0.05, ** p < 0.01, *** p < 0.001

We also performed simultaneous depletion of miR-140-5p and PI3K/AKT while administrating apigenin treatment. Initially, the miR-140-5p levels were assessed using qRT-PCR. Strikely, apigenin treatment effectively reversed the reduction in miR-140-5p levels caused by HG treatment. Conversely, treatment with the miR-140-5p inhibitor significant suppressed miR-140-5p expression (Fig. 5A). Furthermore, apigenin treatment significantly suppressed HDAC3 levels, promoted PTEN levels, and repressed the PI3K/AKT pathway. However, interference with miR-140-5p reversed these effects. Notably, the PI3K/AKT inhibitor inhibited the miR-140-5p-induced activation of the PI3K/AKT pathway (Fig. 5B). In cell function experiments, apigenin treatment significantly reduced the HG-induced increase in cell viability, migration ability, and angiogenesis. Conversely, interference with miR-140-5p promoted these cellular functions, which were subsequently reversed by treatment with the PI3K/AKT inhibitor (Fig. 5C–F). Collectively, these findings suggest that apigenin inhibits angiogenesis in a DR cell model by modulating the miR-140-5p/HDAC3-mediated PTEN/PI3K/AKT pathway.

## Discussion

DR is a dietetic complication that poses a significant threat to vision, characterized by the loss of retinal pericytes and abnormal angiogenesis [21, 22]. Despite its prevalence, the precise underlying mechanism of DR remains incompletely understood. In this study, we aimed to investigate the specific mechanism by which apigenin acts in DR using in vitro experiments. Our findings reveal that apigenin effectively inhibits angiogenesis in HG-induced HRMECs by modulating the miR-140-5p/HDAC3-mediated PTEN/PI3K/AKT pathway. Importantly, this study represents the first report on the function and underlying mechanism of apigenin in the context of DR.

Recently, there has been a growing body of research focused on investigating the role of miRNAs in DR. For instance, Zheng Y et al. reported that miR-126 reduces experimental DR by inhibiting endothelial cell proliferation and migration through its targeting of PLK4 [23]. Similarly, miR-135b-5p inhibits VHL in DR mice leading to increased expression of HIF1α, and promoting endothelial cell proliferation and angiogenesis [24]. These studies underscore the crucial involvement of miRNAs in DR pathogenesis. However, functional role of miR-140-5p in DR remains unexplored.

The miR-140-5p has been implicated in repressing angiogenesis in various conditions, including ischemic stroke [25], breast cancer [26, 27], intervertebral disk degeneration [6], and laryngeal cancer [7]. In our study, we shed light on the down-regulation of miR-140-5p in DR and demonstrated that its overexpression can suppress the proliferation, migration and, angiogenesis of HG-induced HRMECs. Apigenin, a natural compound known for its low toxicity and beneficial health properties [25], has garnered considerable attention [25]. While apigenin has been shown to possess antiangiogenic properties [10, 11], its specific effects on angiogenesis in the context of DR have remained unclear. In this paper, we elucidate that apigenin inhibits the proliferation, migration, and angiogenesis of HG-inducedHRMECs through the regulation of miR-140-5p.

HDAC3 is known to play a role in cell proliferation, apoptosis, transcriptional repression and negatively regulating tumor-induced angiogenic potential [28]. For instance, Yu H et al. reported that miR-4286 targets HDAC3 and influences mouse osteogenesis and angiogenesis, there by mitigating alcohol-induced bone loss in mice [29]. Furthermore, the miR-326-HDAC3 feedback loop has been shown to regulate invasive, tumorigenic, and angiogenic responses to anticancer drugs [30]. These findings suggest that miRNAs can target HDAC3 to modulate angiogenesis. In this study, we established a targeting relationship between miR-140-5p and HDAC3. Furthermore, we revealed that HDAC3 suppresses PTEN expression and activates the PI3K/AKT pathway, thereby promoting proliferation, migration, and angiogenesis of HRMECs in the context of DR.

In conclusion, we demonstrate that miR-140-5p is down-regulated in DR and can be induced by apigenin treatment. Apigenin exerts its inhibitory effects on proliferation, migration, and angiogenesis in HG-inducted HRMECs through the upregulation of miR-140-5p, PTEN, and subsequent inhibition of the PI3K/AKT pathway. These results suggest that apigenin holds promise as a potential therapeutic agent for the treatment of DR, offering new avenues for clinical intervention.

It is important to note that this study primarily focused on investigating the mechanism and function of apigenin using in vitro cell experiments. Therefore, further studies are warranted to validate these findings in an in vivo setting.

## Supplementary Information

**Additional file 1.**

## Data Availability

All data generated or analysed during this study are included in this published article and the Supplementary information files.

## Abbreviations

DR: Diabetic retinopathy
HG: High glucose
VEGF: Vascular endothelial growth factor
miRNAs: MicroRNAs
HDAC 3: Histone deacetylase 3
HRMECs: Human retinal microvascular endothelial cells
qRT-PCR: Quantitative real-time PCR
ChIP: Chromatin immunoprecipitation
ANOVA: One-way analysis of variance
