# Supplementary material for: Apigenin inhibits angiogenesis in retinal microvascular endothelial cells through regulating of the miR-140-5p/HDAC3-mediated PTEN/PI3K/AKT pathway
Source: BMC Ophthalmol. 2023 Jul 6;23:302. doi: 10.1186/s12886-023-03046-5 (PMC10326941; doi:10.1186/s12886-023-03046-5)
Supplement: Supplementary file 1 — Additional file 1. [file 12886_2023_3046_MOESM1_ESM.zip › ADDITIONAL FILE/blots (uncropped).pdf]

Fig3B

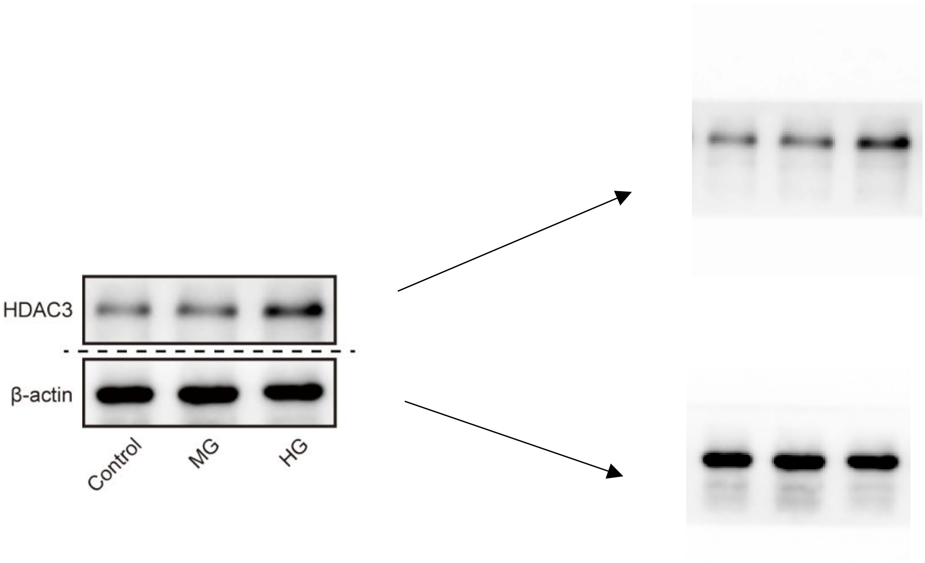
HDAC3
β-actin
Control
MG
HG

Fig3F

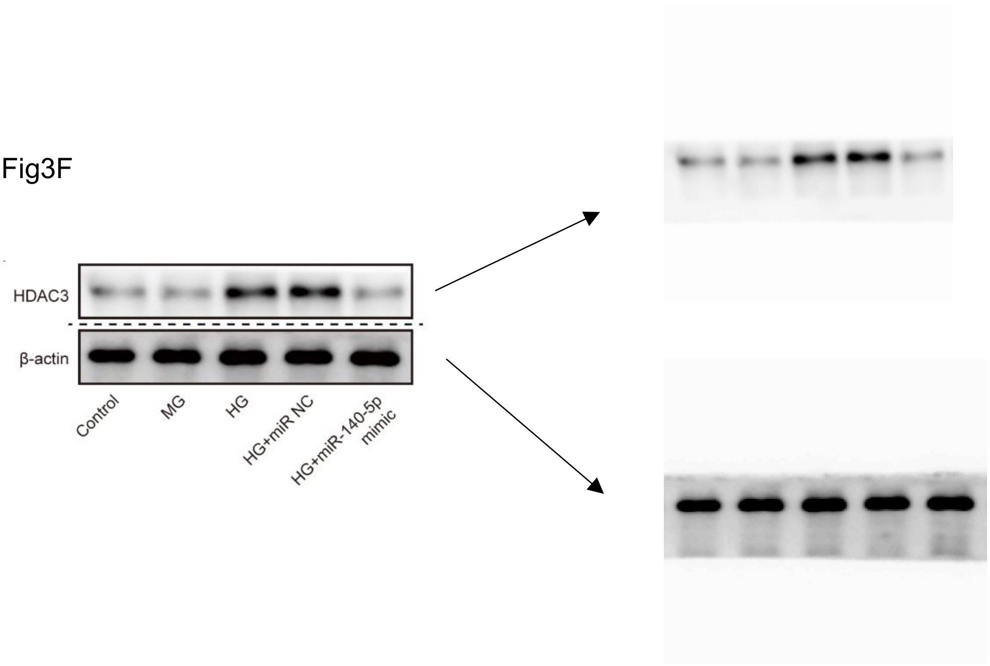
HDAC3
β-actin
Control
MG
HG
HG+miR NC
HG+miR-140-5p mimic

Fig4D

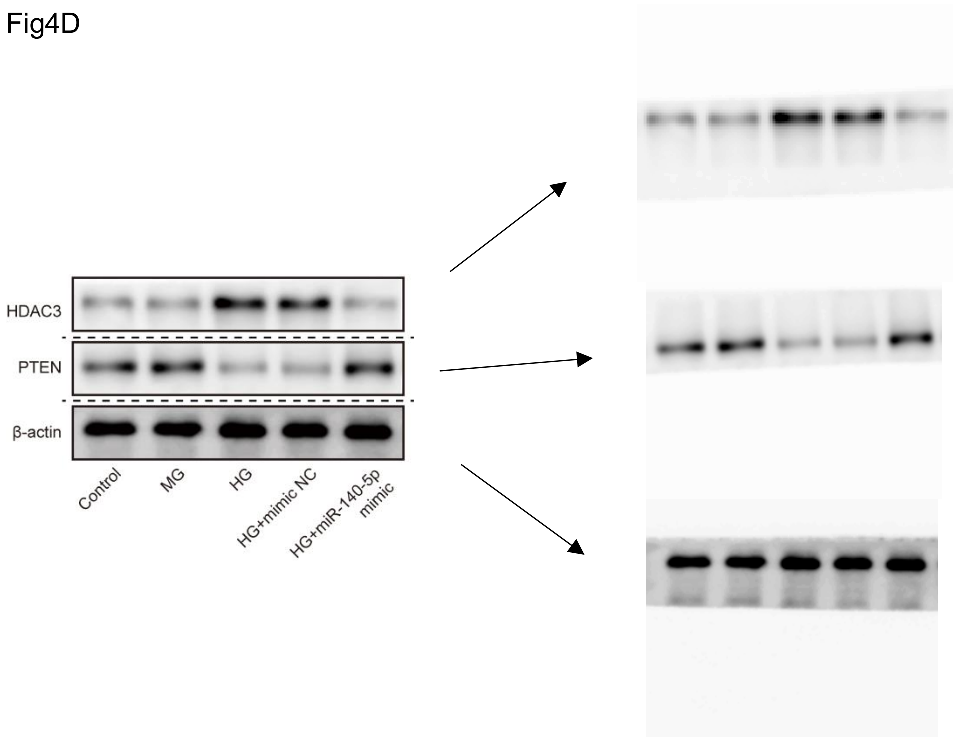
HDAC3
PTEN
β-actin
Control
MG
HG
HG+mimic NC
HG+miR-140-5p mimic

Fig4E

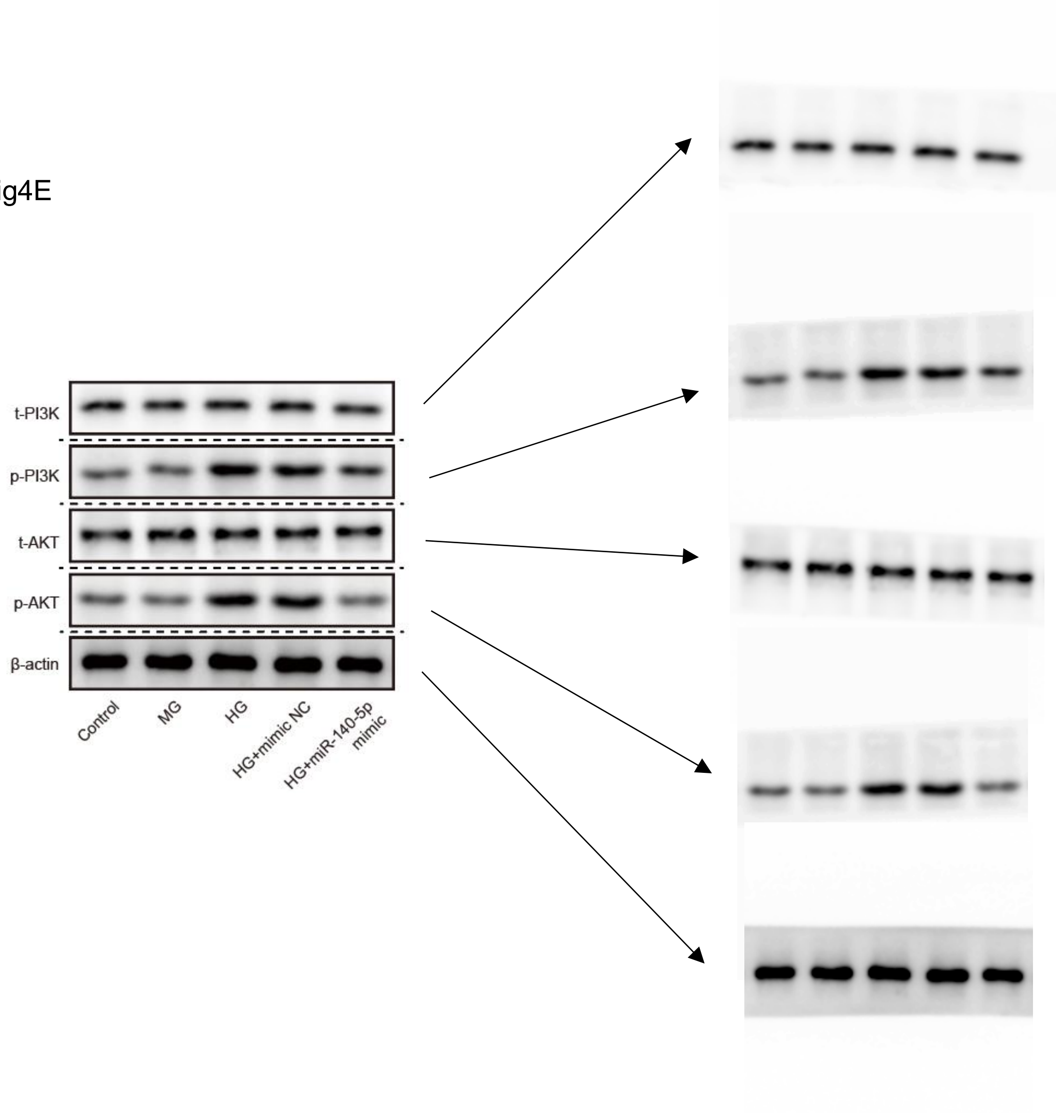
t-PI3K
p-PI3K
t-AKT
p-AKT
β-actin
Control
MG
HG
HG+mimic NC
HG+miR-140-5p mimic

Fig5B

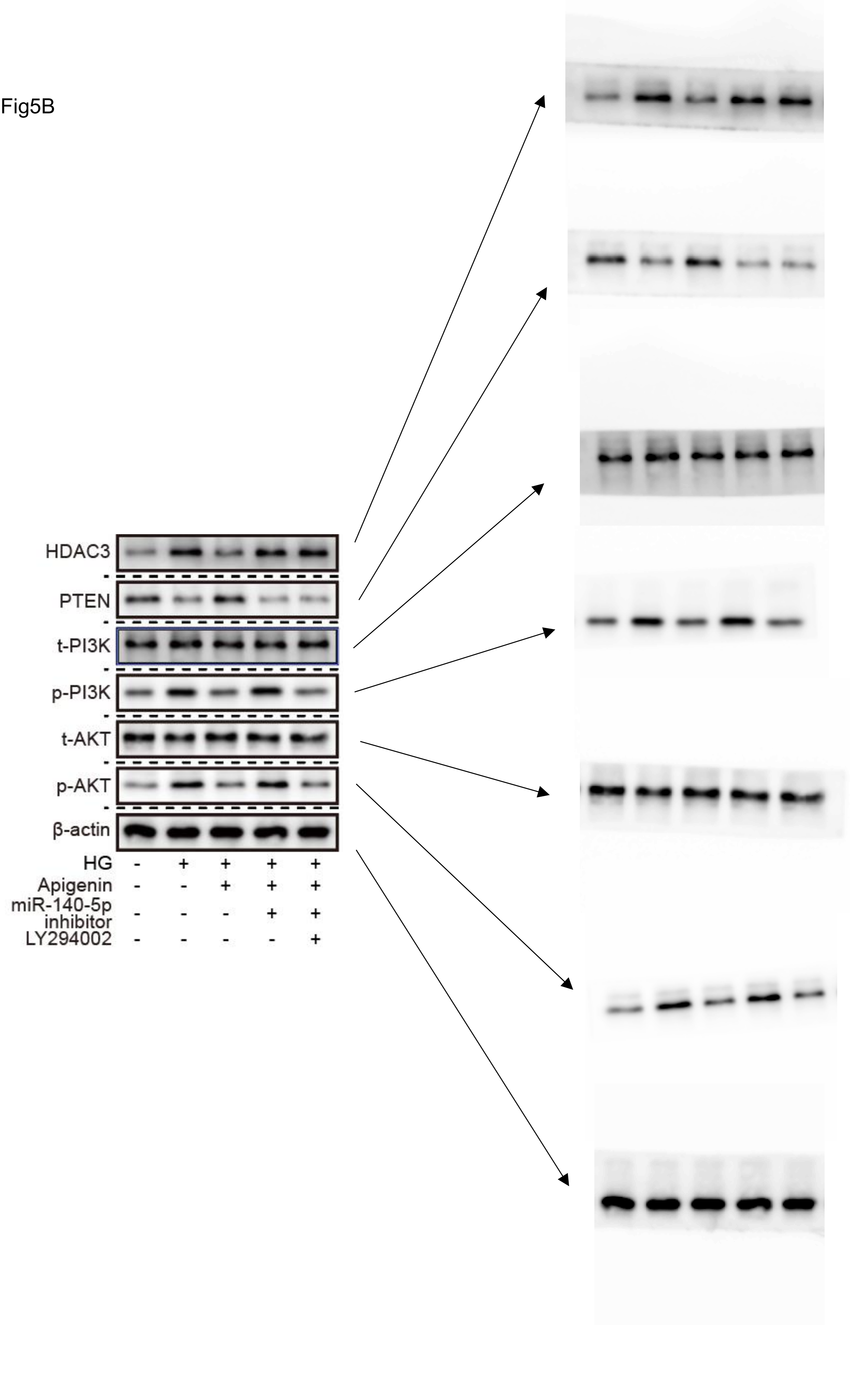

HDAC3
PTEN
t-PI3K
p-PI3K
t-AKT
p-AKT
β-actin
HG - + + + +
Apigenin - - + + +
miR-140-5p inhibitor - - - + +
LY294002 - - - - +
